# Water First Aid Is Beneficial In Humans Post-Burn: Evidence from a Bi-National Cohort Study

**DOI:** 10.1371/journal.pone.0147259

**Published:** 2016-01-25

**Authors:** Fiona M. Wood, Michael Phillips, Tom Jovic, John T Cassidy, Peter Cameron, Dale W. Edgar, Steering Committee of the Burn Registry of Australia and New Zealand (BRANZ)

**Affiliations:** 1 Burn Service of Western Australia, State Adult Burn Unit, Fiona Stanley Hospital, Murdoch, Western Australia, Australia; 2 Burn Injury Research Unit, University of Western Australia, Crawley, Western Australia, Australia; 3 Fiona Wood Foundation, Murdoch, Western Australia, Australia; 4 Perkins Institute of Medical Research, Royal Perth Hospital, Perth and University of Western Australia, Crawley, Western Australia, Australia; 5 University of Edinburgh, Edinburgh, Scotland, United Kingdom; 6 Burn Service of Western Australia, Burn Unit, Royal Perth Hospital, Perth, Western Australia, Australia; 7 James Connolly Memorial Hospital, Blanchardstown, Dublin, Ireland; 8 Department of Epidemiology and Preventive Medicine, Monash University, Melbourne, Victoria, Australia; 9 Burn Injury Research Node, The University of Notre Dame Australia, Fremantle, Western Australia, Australia; Robert Bosch Hospital, GERMANY

## Abstract

**Introduction:**

Reported first aid application, frequency and practices around the world vary greatly. Based primarily on animal and observational studies, first aid after a burn injury is considered to be integral in reducing scar and infection, and the need for surgery. The current recommendation for optimum first aid after burn is water cooling for 20 minutes within three hours. However, compliance with this guideline is reported as poor to moderate at best and evidence exists to suggest that overcooling can be detrimental. This prospective cohort study of a bi-national burn patient registry examined data collected between 2009 and 2012. The aim of the study was to quantify the magnitude of effects of water cooling first aid after burn on indicators of burn severity in a large human cohort.

**Method:**

The data for the analysis was provided by the Burn Registry of Australia and New Zealand (BRANZ). The application of first aid cooling prior to admission to a dedicated burn service, was analysed for its influence on four outcomes related to injury severity. The patient related outcomes were whether graft surgery occurred, and death while the health system (cost) outcomes included total hospital length of stay and admission to ICU. Robust regression analysis using bootstrapped estimation adjusted using a propensity score was used to control for confounding and to estimate the strength of association with first aid. Dose-response relationships were examined to determine associations with duration of first aid. The influence of covariates on the impact of first aid was assessed.

**Results:**

Cooling was provided before Burn Centre admission for 68% of patients, with at least twenty minutes duration for 46%. The results indicated a reduction in burn injury severity associated with first aid. Patients probability for graft surgery fell by 0.070 from 0.537 (13% reduction) (*p* = 0.014). The probability for ICU admission fell by 0.084 from 0.175 (48% reduction) (*p*<0.001) and hospital length of stay (LOS) fell by 2.27 days from 12.9 days (18% reduction) (*p* = 0.001). All outcomes except death showed a dose-response relationship with the duration of first aid. The size of burn and age interacted with many of the relationships between first aid and outcome and these are described and discussed.

**Discussion & Conclusion:**

This study suggests that there are significant patient and health system benefits from cooling water first aid, particularly if applied for up to 20 minutes. The results of this study estimate the effect size of post-burn first aid and confirm that efforts to promote first aid knowledge are not only warranted, but provide potential cost savings.

## Introduction

It is an accepted teaching that earlier intervention with evidence based treatments for burn injuries is associated with improved outcomes and lower complication and mortality rates [1, 2]. Within the pre-hospital period, first aid is thought to be an important measure to minimise consequences of injury but there is a lack of reported human data to support this. The ability to understand the effect of first aid in human patients is hindered by the fact that first aid teachings are not consistent around the world [3, 4], can be influenced by first aider characteristics [5, 6] and compliance with application of appropriate first aid is reported above 50%[3, 7], with many observational study findings far below this.

The Emergency Management of Severe Burns guidelines stipulate that ‘adequate’ first aid for burns involves the application of cool running water for a minimum of 20 minutes within the first three hours of injury [8]. Animal studies have confirmed these parameters showing that the immediate application of cool running water was associated with faster re-epithelialisation and reduced scarring [3]. The optimum duration of water cooling in human patients is unknown. Excessive cooling of burn patients, resulting in hypothermia, was reported to be associated with adverse outcomes including disordered clotting and increased mortality [9]. Studies of patients suggest that the extent of surgical intervention required and length of hospital stay may be reduced by first aid [10, 11]. The relationship between first aid and post-burn mortality has not been investigated. Such studies are complicated by difficulties with accurately recording the timeliness, duration and method of water application prior to medical care. There have been no randomised controlled human trials for both ethical and logistical reasons.

The aim of this study was to estimate the magnitude of the association between water cooling first aid on short term post-burn outcomes in humans using data acquired from the Burns Registry of Australia and New Zealand (BRANZ).

The subsequent hypotheses were that water cooling first aid is associated with a reduction in:

1. the risk of requiring intensive care unit (ICU) admission;
2. the risk of skin grafting surgery;
3. the length of stay (LOS) in the acute hospital environment; and,
4. mortality risk.

## Method

### Study Design

This was a retrospective analysis of a cohort of patients using prospectively collected registry data. The data was compiled between 1st July 2009 and the 30th March 2012. The BRANZ registry results from a collaboration between the Australian and New Zealand Burn Association and Monash University, Melbourne. The database includes data acquired from a total of 2,897 admitted patients aged 16 years or more in the study period.

The de-identified data was collected by trained nurses at each Burn Centre and then submitted to BRANZ. Patients were recorded if they were admitted to a Burn Centre within 28 days of the injury [12].

### Data Analysis

The available data included the patient’s age, gender and injury details (total percentage of body surface area injured (TBSA), mechanism/agent of injury and anatomical location of burn wounds). The short term outcomes investigated were determined during the planning of the study and included: a) ICU admission; b) graft surgery; c) hospital LOS; and, d) in-hospital mortality. The duration of water cooling first aid was recorded (in minutes) as provided: a) at the scene; b) by paramedics; c) in an interim hospital; d) in an emergency department. The summation of this pre-hospital information was used in conjunction with the date and time of injury and admission to estimate the duration of first aid provision. Patients with the following characteristics were excluded: a) inhalation or airway injury; b) electrical injury; c) chemical burns; d) non-thermal causes; and, e) cold injury.

Data were described as counts, percentages and median with inter-quartile range as appropriate. The independent variables of interest were water first aid and duration of application (minutes) with no recorded water first aid as the reference. For univariable analysis Pearson chi-squared was used for any first aid and Mann-Whitney U test was used for duration. Associations between the duration of water first aid and the outcomes showed clear indication of non-linearity. We decided to categorise the first aid cooling duration data into five categories (none, 1–9 mins, 10–19 mins, 20–39 mins and 40 or more mins) despite the well-documented problems of categorizing continuous data because it allowed non-linearity to become apparent.

For ICU admission, grafting surgery and mortality a multivariable logistic regression was used and for LOS a multivariable truncated negative binomial regression model was used. Patients who died in hospital were excluded from all analyses other than death. Multivariable analyses were controlled for age, gender and TBSA. Restricted cubic spline transformations were used to examine non-linearity and interaction terms were incorporated when necessary. All models were adjusted using robust estimation methods to allow for differences in pre-hospital systems and clustering within Australian States and New Zealand was applied. All analyses used bootstrap estimation to control for over-fitting. The logistic regression models were assessed for goodness-of-fit using the method of Hosmer and Lemeshow [13].

To facilitate interpretation of the non-randomised treatment effect we used methods based upon proximity scores with variables associated with first aid treatment used as the basis for proximity score estimation. Our analysis followed the recommendations of Stuart [14] and were limited to doubly robust methods [15]. For any water first aid we used proximity score matching to minimise confounding by observed and unobserved variables. For estimation of the dose-response relationship between outcomes and the duration of first aid we used augmented inverse-probability weighted estimation.

A *p* value less than 0⋅05 was regarded as statistically significant and all statistical tests were two-tailed. Data were analysed using Stata (V.13.1, StataCorp, College Station, Tx, USA).

### Ethics

Ethics approval for the study was provided by the Royal Perth Hospital Human Research Ethics Committee (Approval number EC 2012/092) prior to final approval by the ANZBA-Monash BRANZ Steering Committee. Data were supplied de-identified from the BRANZ and was not re-identifiable by the investigators. Ethics approval was provided with the understanding that individual patient consent was not to be sought because the data were not identifiable, the cohort was large and distributed across two countries and multiple territorial jurisdictions.

## Results

### Sample Description

Of the 2,897 records, 577 (20%) were excluded due to the presence of inhalation injury or because the cause of injury was not appropriate for water first aid. For the remaining 2,320 patients, 68% had a period of water cooling recorded before admission to the Burn Centre. Of those, 97% received water within three hours of the injury.

Data were complete for age and first aid provision. There was missing data for: length of hospital stay (n = 7; 0∙3%); ICU admission (n = 6; 0∙2%); graft surgery (n = 23; 1.49%); TBSA (n = 79; 3.41%); and duration of first aid (n = 196; 8.45%). There was no association between missing duration of first aid and any of the outcomes or covariates. There was a significant association with missing TBSA and any first aid (*p* = 0.012), duration of first aid (*p*<0.001), graft surgery (*p*<0.001), ICU admission (*p* = 0.027), and hospital LOS (*p*<0.001).

The median age of the study sample was 36 years (IQR = 24–51) and 75% were males (Table 1). The median time to admission to a Burn Centre was 5.2 hours (IQR = 1.8–13) with 49% directly admitted to the Burn Centre. More than half were injured in their own or another home (64%). The median TBSA of the sample was 5.5% (IQR = 3–10) with a burn of ≥25% TBSA in 5% of cases. The median hospital LOS was 7 days (IQR = 3–14) and ICU LOS 2 days (IQR = 1–5.4). Eighty two percent of patients were recorded to have multiple body sites of injury. The largest proportion was to the upper limbs (67%) with hand involvement in 80%, followed by lower limbs (49%), the face (39%) and trunk (27%). There were 24 deaths (1.0%). Mortality was highest in those with larger TBSA injuries (Mann-Whitney test, Z = -2.15, *p* = 0.032).

**Table 1:** Description of the sample.

**Variable**	**Median**	**IQR**	**N**
Age (yrs)	36	24–51	2320
TBSA (%)	5.5	3–10	2241
Hospital LOS (days)	7	2.96–13.8	2313
ICU LOS (days)	2	1–5.4	261
Time to admission (hrs)	5.2	1.8–13	2313
**Variable**		**%**	**N**
Gender	Male	75.2	2,319
Burn injury cause	Contact	7.93	184
	Scald	28.8	667
	Flame	56.6	1,313
	Friction	5.69	132
	Radiant heat	1.03	24
Place of injury	Residential	1,417	63.8
	Commercial	309	13.9
	Street or highway	217	9.77
	Recreational/sporting	178	8.01
	Farm	57	2.57
	Other location	43	1.94
	Not recorded	99	
Part of body injured	Face	58	3.18
	Torso	58	3.18
	Upper limb(s)	122	6.68
	Lower limb(s)	96	5.26
	Multiple sites	1,492	81.7
	Not recorded	494	

### Factors Influencing Water First Aid Provision

Sixty eight percent (68%) of people received water first aid at some point before admission to a Burns Centre (Table 2). However, cool water first aid for at least 20 minutes within three hours of injury was recorded in only 46% of cases. The majority of burn patients received first aid at the scene of the accident (91%) and 19% received first aid on a second occasion before admission.

**Table 2:** Description of first aid provision.

**Water first aid**	**N (%)**		**Total**	
**Any water**	1,540 (68.4)		2,320	
**Duration of water first aid (mins)**	**Later than 3 hrs**	**Within 3hrs**	**Total**	
Water for 1–9 mins	7 (0.49)	286 (20.0)	293 (20.5)	
Water for 10–19 mins	13 (0.91)	351 (24.5)	364 (25.4)	
Water for 20–39 mins	12 (0.84)	525 (36.7)	537 (37.5)	
Water for 40+ mins	10 (0.70)	228 (15.9)	238 (16.6)	
Total	42 (2.93)	1,390 (97.1)	1,432 (100)	
**Agent of injury**	**Water within 3hrs (%)**		**Total**	
Contact	85 (46.2)		184	
Scald	505 (75.7)		667	
Flame	936 (71.3)		1,313	
Friction	9 (6.82)		132	
Radiant Heat (no contact)	5 (20.8)		24	
	**No water first aid**		**Water within 3 hours**	
	**Median**	**IQR**	**Median**	**IQR**
Age (yrs)	38	25–55	34	23–49
TBSA (%)	5	2–10	6	3–12
**Place of injury**	**Water within 3hrs (%)**		**Total**	
Own home	919 (74.9)		1,227	
Other residence	137 (80.9)		157	
Residential institution	9 (45.0)		20	
School/educational institution	10 (47.6)		21	
Sports facility	8 (80.0)		10	
Commercial	244 (80.5)		303	
Street or highway	62 (29.7)		209	
Place for recreation	88 (54.0)		163	
Farm	43 (79.6)		54	
Not recorded	54 (54.6)		99	

There was a significant association between the provision of first aid and the type of burn injury with 76% of scalds and 71% of flame injuries receiving the intervention but only 46% of contact burns, 20% of radiant heat burns and 7% of friction burns (χ24 = 332.5, *p*< 0.001). There was also a significant association between first aid provision and age with younger people more likely to be treated (median age 34 vs. 38, Z = 4.15, *p*<0.0001). The size of burn was associated significantly with application of first aid, with large burns more likely to be treated (median TBSA 6% vs. 5%, Z = -4.62, *p*<0.0001). There was no association with gender (64% of women vs. 67% men, χ22 = 1.92, *p* = 0.17). Place of injury was significantly associated with first aid provision (χ25 = 211, *p* < 0.0001) with >70% of burns occurring in homes, sporting venues, commercial locations and farms being given first aid whereas it was significantly less likely to be provided if the injury occurred in a residential institution (45%), school or other institution (48%), on a street or highway (29%) or a place of recreation (51%). Fire type was also significantly associated with first aid provision with 75% of those involved in explosions being given first aid compared with 52% of those in a vehicle fire and 56% of those in a house fire (*p* < 0.0001).

The duration of first aid ranged from 2 to 75 minutes, with a median duration of 25 mins (IQR: 15–25). There was no association between the duration of water first aid and gender (Z = 0.130, *p* = 0.90), age (Spearman’s rho [ρS] = -0.014, *p* = 0.60) or type of fire (χ23 = 5.20, *p* = 0.82). Duration of first aid correlated significantly with time to admission with patients who had a longer time to admission receiving a greater duration of water cooling (ρS = -0.085, *p*<0.0001). There was a significant association with place of burn with those in their own home likely to be provided with first aid for a longer period than those injured elsewhere. Those in a residential institution, school or other institution, on a street or highway or farm were likely to be provided for a shorter duration (χ25 = 149, *p* = 0.029). There was also an association between duration of first aid and cause of burn (χ24 = 198, *p* = 0.0001, Kruskal-Wallis test). However when the analysis was limited to burns with first aid provided there was no difference in duration between the causes of injury (χ23 = 0.132, *p* = 0.988, Kruskal-Wallis test) so that the difference shown related to whether first aid is provided rather than the time of provision.

### Influence of First Aid on Post-burn Outcomes

The association between each of the selected outcomes and the provision of timely water first aid is shown in Table 3 without covariate adjustment. We observed no association with ICU admission (OR = 0.77, *p* = 0.35) or wound repair surgery (OR = 0.75, *p* = 0.17). For death there was a significant association (OR = 0.30, *p* = 0.013). There was a significant reduction in hospital length of stay associated with provision of water first aid: a reduction of 0.24 days or 22% (truncated negative binomial regression, *p*<0.001).

**Table 3:** Results of bootstrapped analyses estimating the influence of water first aid on outcomes (without adjustment for covariates).

**First aid intervention or covariate**	**Outcome**	**Odds Ratio**	**95% CI for OR**^***2***^		***p***
			**LCL**	**UCL**	
Water first aid within 3 hours	**ICU admission**	0.770	0.453	1.32	0.345
Constant		0.146	0.080	0.264	<0.001
Water first aid within 3 hours	**Wound repair surgery**	0.745	0.485	1.11	0.17
Constant		1.50	0.739	3.03	0.26
Water first aid within 3 hours	**Death**	0.300	0.102	0.836	0.013
Constant		0.020	0.010	0.037	<0.001
	**Outcome**	**LOS**	**95% CI**		***p***
			**LCL**	**UCL**	
Water first aid within 3 hours	**Hospital LOS**	-0.243	-0.351	-0.134	<0.001
Constant	(days)	2.39	2.30	2.48	<0.001
**Duration of water provision**	**Outcome**	**Odds Ratio**	**95% CI**		***p***
			**LCL**	**UCL**	
No water provided	**ICU admission**	1 (ref.)			
Water for 1–9 mins		0.559	0.332	1.13	0.052
Water for 10–19 mins		0.511	0.263	0.898	0.033
Water for 20–39 mins		0.864	0.391	1.51	0.72
Water for 40+ mins		1.13	0.748	3.33	0.73
Constant		0.151	0.081	0.279	<0.001
No water provided	**Wound repair surgery**	1 (ref.)			
Water for 1–9 mins		0.692	0.496	1.84	0.24
Water for 10–19 mins		0.665	0.440	1.29	0.11
Water for 20–39 mins		0.539	0.309	0.939	0.036
Water for 40+ mins		1.59	0.378	2.61	0.41
Constant		1.55	0.764	3.16	0.22
No water provided	**Death in hospital**	1 (ref.)			
Water for 1–9 mins		0.168	0.121	0.328	<0.001
Water for 10–19 mins		0.137	0.116	0.238	<0.001
Water for 20–39 mins		0.461	0.415	0.541	0.004
Water for 40+ mins		0.211	0.063	0.541	0.006
Constant		0.021	0.014	0.031	<0.001
No water provided	**Hospital LOS**	2.39	2.30	2.49	<0.001
Water for 1–9 mins	(days)	-0.377	-0.545	-0.209	<0.001
Water for 10–19 mins		-0.358	-0.514	-0.201	<0.001
Water for 20–39 mins		-0.182	-0.319	-0.045	0.009
Water for 40+ mins		-0.073	-0.254	0.108	0.43

There was an indication of significant non-linear associations with the duration of first aid for all outcomes. For ICU admission the OR was significant for water duration of 10–19 minutes duration (OR = 0.51, *p* = 0.033). For wound repair surgery the OR was significant for water duration of 20–39 minutes duration (OR = 0.54, *p* = 0.036). For death all durations were associated with a significant reduction in risk and for LOS all durations less than 40 minutes were associated with a reduction and a pattern which may reflect a diminishing exposure-response effect (Table 3).

**Fig 1:**
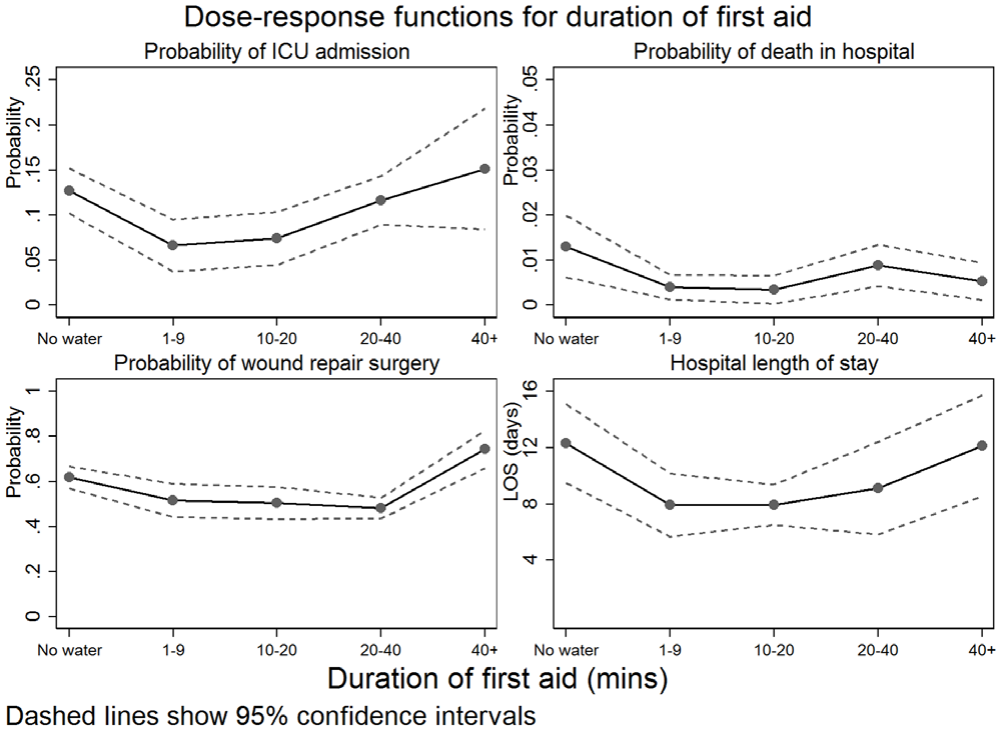
Comparison of dose response to first aid for each outcome variable.

Two hundred and fifty patients were admitted to ICU (11%). Proximity score matching analysis indicated that water first aid was significantly associated with a reduction in the probability of ICU admission (*p*<0.001). A similar analysis showed that the duration of water first aid significantly reduced ICU admission probability for durations less than 10 minutes (*p* = 0.023) (Table 4 and Fig 1).

**Table 4:** Results of bootstrapped analysis using propensity score (PS) matching for any water and PS weighting for duration of water.

**Outcome**	**Estimate**	**Probability of outcome**	**95% CI**		***p***
			**LCL**	**UCL**	
**ICU admission**	Any water first aid vs. none	-0.084	-0.125	-0.043	<0.001
	No water first aid	0.175	0.135	0.215	<0.001
**Wound repair surgery**	Any water first aid vs. none	-0.070	-0.125	-0.014	0.014
	No water first aid	0.537	0.507	0.567	<0.001
**Death**	Any water first aid vs. none	-0.0031	-0.010	0.004	0.39
	No water first aid	0.0093	0.0029	0.0157	0.005
**Difference in hospital LOS** (days)	Any water first aid vs. none	-2.27	-3.61	-0.931	0.001
	No water first aid	12.9	11.7	14.2	<0.001
**Outcome**	**Estimates by duration of water provision**				
**ICU admission**	No water	1(ref)			
	1–9 mins	0.487	0.276	0.766	0.023
	10–19 mins	0.551	0.354	0.899	0.057
	20–39 mins	0.907	0.698	1.37	0.58
	40+ mins	1.23	0.678	2.36	0.44
**Wound repair surgery**	No water	1(ref)			
	1–9 mins	0.657	0.425	0.927	0.030
	10–19 mins	0.629	0.461	1.11	0.017
	20–39 mins	0.573	0.461	0.882	<0.001
	40+ mins	1.78	1.01	2.65	0.028
**Death**	No water	1(ref)			
	1–9 mins	0.299	0.164	0.651	0.005
	10–19 mins	0.260	0.119	0.577	0.005
	20–39 mins	0.673	0.368	1.29	0.30
	40+ mins	0.401	0.121	1.05	0.12
	**Duration of water provision**	**Coefficient**	**LCL**	**UCL**	***p***
**Hospital LOS** (days)	No water	12.3	9.51	15.1	<0.001
	1–9 mins	7.92	5.67	10.2	<0.001
	10–19 mins	7.95	6.51	9.38	0.040
	20–39 mins	9.13	5.83	12.4	0.94
	40+ mins	12.1	8.55	15.7	0.77

*1* Propensity scores based upon: Age, TBSA, Gender, Australian State or New Zealand, Cause of injury, Place of injury, Injury date, Explosion, Fire type, Time to admission, number of body parts injured.

Sixty-nine percent (69%) of the patient sample were recorded as undergoing a procedure in theatre and 55% of the total number of theatre procedures indicated that skin grafting (reconstructive) surgery was required (n = 837). Proximity score matching showed that the application of water first aid significantly reduced the probability of grafting surgery (*p* = 0.014). There was a significant reduction in the probability of graft surgery for all times less than 40 minutes (*p*≤0.03) but a significant increase in probability for duration of 40 minutes or more (*p* = 0.028).

The geometric mean total LOS was 6.60 days (95% CI: 6.32–6.89). The application of water first aid was significantly associated with a reduction of 2.27 days (*p* = 0.001). There was a significant reduction of LOS for water first aid up to 20 minutes but not for longer times (*p*≤0.04).

The application of any water first aid was not significantly associated with a reduced risk of death (*p* = 0.39) but an analysis of duration of first aid did indicate a dose-response relationship with a significant reduction in risk for duration below 20 minutes (*p* = 0.005).

### Influence of Covariates on Post-burn Outcomes

The influence of independent covariates was examined using interaction terms in the robust regression analyses. Non-linear associations with the outcomes were examined using restricted cubic spline transformations. The results are shown in Table 5.

**Table 5:** Results of multivariable regression analysis to examine linearity of association for each outcome using restricted cubic splines and interaction terms for continuous independent variables.

**Outcome**	**Odds Ratio**	**95% CI for OR**		***p***
**Wound repair surgery**		**LCL**	**UCL**	
No water first aid	1 (Ref.)			
Water first aid provided	0.517	0.290	0.921	0.025
TBSA	1.06	1.00	1.11	0.032
TBSA*Water interaction	1.04	1.01	1.07	0.003
Age	1.02	1.01	1.02	<0.001
**ICU admission**				
No water first aid	1 (Ref.)			
Water first aid provided	0.421	0.221	0.803	0.009
TBSA (mean standardised)	2.26	1.84	2.78	<0.001
TBSA (spline transformation 1)	1.32	1.15	1.51	<0.001
TBSA*Water interaction	1.03	0.999	1.06	0.062
Age	1.01	1.00	1.01	<0.001
**Death**				
No water first aid	1 (Ref.)			
Water first aid provided	0.042	0.002	0.744	0.031
TBSA	1.09	1.05	1.13	<0.001
Age (mean standardised)	2.25	1.33	3.80	0.002
Age (spline transformation 1)	1.15	0.756	1.76	0.508
Age (spline transformation 2)	1.87	1.07	3.28	0.028
Age*Water interaction	1.03	1.002	1.06	0.035
**Hospital LOS** (days)	**Coefficient**	**95% CI**		***p***
		**LCL**	**UCL**	
No water first aid	0 (ref.)			
Water first aid provided	-0.113	-0.306	0.080	0.250
TBSA (mean standardised)	0.358	0.268	0.449	<0.001
TBSA (spline transformation 1)	0.160	0.124	0.196	<0.001
TBSA (spline transformation 2)	-0.049	-0.118	0.021	0.170
TBSA*Water interaction	0.020	0.008	0.032	0.001
Age (mean standardised)	0.238	0.220	0.256	<0.001
Age (spline transformation 1)	-0.017	-0.059	0.026	0.445
Age (spline transformation 2)	0.013	-0.011	0.037	0.285
Age (spline transformation 3)	-0.048	-0.129	0.032	0.238
Age*Water interaction	-0.164	-0.250	-0.078	<0.001
Age*Water (spline transformation)	-0.099	-0.157	-0.041	0.001
Constant	2.20	2.02	2.38	<0.001

The results of the covariate analyses show that both TBSA and age influence all of the outcomes.

**Fig 2:**
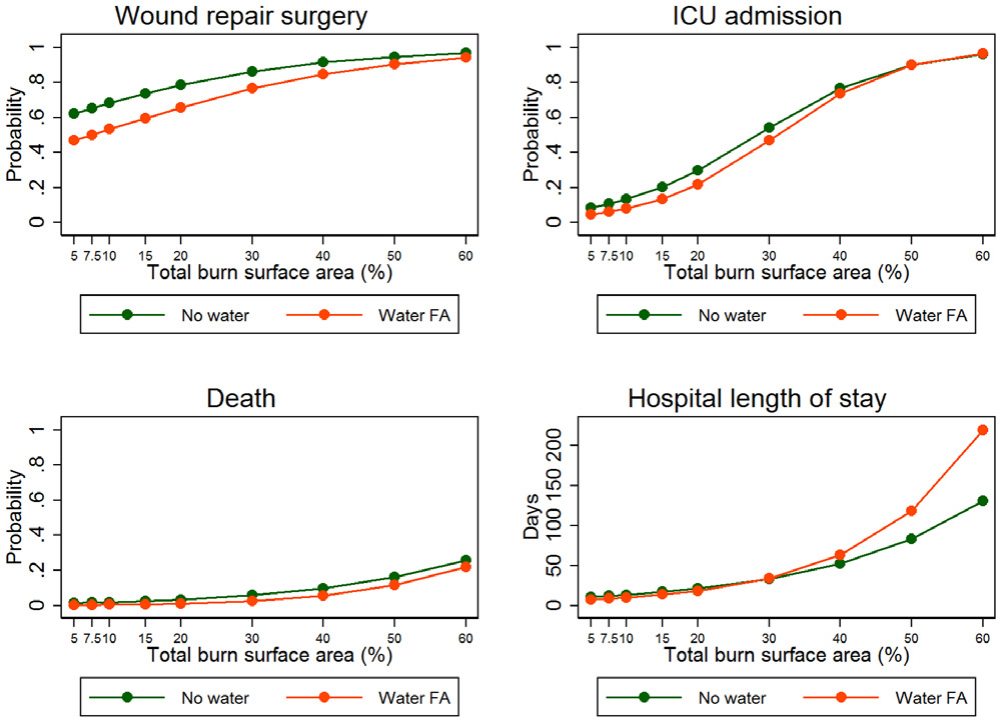
Association between TBSA and first aid for each outcome variable.

For wound repair surgery there is a linear increase in probability with increasing age and with increasing TBSA. In addition there is an interaction between water first aid and TBSA which indicates that the increase in probability is associated with a stronger association with TBSA for those given water first aid. The result indicates that for this outcome the effect of first aid is greater in smaller burns (Fig 2).

For ICU admission there is a linear increase in probability with increasing age and a non-linear increase in probability with TBSA. There is also a significant positive interaction between first aid and TBSA indicating that the slope is greater for those given first aid. These results are consistent with a greater benefit in small and, particularly, medium sized burns (Fig 2).

For death there is a significant linear association with TBSA. There is also a significant but non-linear association with age and a significant positive interaction between age and first aid suggesting that the benefit of first aid with respect to death is greater at older ages.

There is a powerful and complex relationship between an increase in both age and TBSA and hospital length of stay. Both of these variables also influence the effect of water first aid on LOS as is indicated by a significant interaction for TBSA and a non-linear interaction for age. The associations with age and TBSA are both positive, as might be expected. For age, the interaction analysis and transformations indicated that as age increases, so too does LOS. However, this interaction is reduced by water first aid. For TBSA, LOS increases as TBSA increases in a non-linear manner. The interaction term is positive which suggests that larger burns result in a *longer* LOS after first aid (Fig 2).

## Discussion

This large human cohort study showed that water cooling was associated with a significant reduction in risk of: ICU admission and wound repair surgery but possibly not mortality. Further, the study showed water cooling reduced the average hospital stay by more than two days.

The study outcomes with respect to post-burn benefits after water cooling first aid were consistent with the early inferences of Sir Archibald McIndoe after the World War II [16]. This is the first large scale human study to quantify those benefits and demonstrate a dose response relationship associated with the duration of water cooling in some outcomes. The results of this study suggest that water cooling for 20–25 minutes in the first three hours after acute burn injury should be a required standard by pre-hospital and hospital health care providers and a key education point in community burn injury minimisation campaigns. We base this recommendation on the generality of the results which suggest that there is little benefit beyond 20 minutes and there is a possibility of harm at prolonged durations.

It is unlikely that our results are a consequence of confounding as they are based upon proximity matching with eleven demographic and circumstantial covariates which precede or are co-terminal with the injury. The multivariate analyses add additional support as they demonstrated an interaction between TBSA and water first aid for all outcomes except death and interactions between water first aid and age for death and length of stay.

Further, cooling for more than 40 minutes in total does not appear to have a significantly positive effect and may be detrimental. This result was similar to the results from a porcine study [17]. It is postulated that the negative effects of long duration cooling may be related to a drop in core temperature as was possibly evident in larger burns in this study. This interpretation is supported by the apparent paradox of the interaction between TBSA and water first aid which shows a longer length of stay for larger burns for those who received first aid. There are two possible explanations for this pattern. The first is that first aid is aiding patients with large burns to survive and survivors are associated with a longer length of stay. (Note that patients who died in hospital were excluded from this analysis for all outcomes except death.) The other explanation is that first aid has side-effects which result in longer length of stay and a possible mechanism is that the pattern reflects an effect of hypothermia.

While this study strongly supports the beneficial application of cool water first aid, there are notable limitations which relate to the type of study and use of registry data. The multicentre nature of the data collection improves external validity but may introduce bias due to geography and a lack of standard protocols for transfer and pre-hospital practices. For this reason all models used robust estimation of standard errors based upon geographic clustering and by including geographic location in the proximity score analyses. However bias may still be present as a consequence of associations between water cooling and unobserved factors such as socioeconomic status; co-morbidity; and drug and alcohol use.

Although the debate concerning statistical control of bias from confounding exists it is important not to forget the observation of Sir Austin Bradford Hill that if there is evidence of a benefit from an action that is inexpensive and unlikely to cause harm it would be foolish not to act even if it turns out not to be a causal association. That said, on the basis of these findings, a more detailed investigation into the effectiveness of first aid is indicated.

There are a number of areas of interest that emerge from this study and prompt ideas for further research to examine the physiological mechanism underlying the benefits caused by cool water first aid. One is to investigate the possible side-effects from hypothermia induced by water cooling. This information was not available for this study and there are a number of these which have been described and investigated as a result of induced hypothermia in the critical care setting [18]. Of primary importance in the burn injury context is the identified effect of cutaneous vasoconstriction and the association with increased infection risk. Another area which has been addressed superficially in this paper is the complexity of interactions between outcomes, water first aid and covariates such as burn severity (as indicated by TBSA) and age. Further detailed analyses are required to provide a more comprehensive description of these results for publication in the future. Another topic which deserves further investigation is to estimate the cost-effectiveness of first aid training and information dissemination. An additional limitation of our study is that we excluded children in order to limit heterogeneity of our sample. A study of paediatric burns patients is necessary so that we can identify likely variations of the impact of first aid where the surface area to body volume ratio is so very different.

## Conclusion

This study has confirmed the magnitude of benefit from first aid after burn and emphasised the parameters of water cooling to achieve a significantly reduced need for surgical intervention, length of stay and ICU admission. Further studies with a larger dataset are needed to confirm or refute the association with risk of death.
